# The association between pain intensity and the prescription of analgesics and non-steroidal anti-inflammatory drugs

**DOI:** 10.1002/j.1532-2149.2011.00107.x

**Published:** 2012-01-19

**Authors:** S Muller, J Bedson, CD Mallen

**Affiliations:** Arthritis Research UK Primary Care Centre, Keele UniversityStaffordshire, UK

## Abstract

**Background:**

It is not known whether general practitioners (GPs) prescribe analgesic medication according to intensity of pain or a hierarchical prescribing regimen.

**Aims:**

The aim of this study was to assess the association of strength of pain-relief medication prescribed by the GP with the strength of previous prescription and pain level.

**Methods:**

The PROG-RES study collected data on pain intensity in 428 patients aged ≥50 years with non-inflammatory musculoskeletal pain during a consultation with their GP. Prescriptions for analgesics and non-steroidal anti-inflammatory drugs (NSAIDs) were identified on the day of the consultation and in the previous year and were classified as basic, moderate or strong analgesic or NSAID. Regression models were used to assess the association of strength of analgesia and prescription of a NSAID with the strength of previous prescription and the level of pain.

**Results:**

The majority of patients were not prescribed medication for their pain at the index consultation, but had such a prescription the previous year. There was an association between strength of analgesic and intensity of pain: more intense pain resulted in a stronger drug. This association was attenuated by adjustment for prescribed analgesia in the previous year. There was no association between intensity of pain and NSAID prescription, but previous NSAID prescription predicted another such prescription.

**Conclusion:**

GPs do not always issue prescriptions for musculoskeletal pain. In cases where a prescription is issued, this is more strongly influenced by previous prescriptions than the patient's pain level. GPs adopt an individualized approach to the treatment of musculoskeletal pain in older adults.

## 1. Introduction

Musculoskeletal pain is one of the most common reasons for consultation with a general practitioner (GP) in the United Kingdom, accounting for around one in every seven GP consultations (Jordan et al., 2010). Ehrlich (2003) suggested the application of the World Health Organisation's (WHO) analgesic ladder for cancer pain (WHO, 1986) to those reporting back pain, and the National Institute for Health and Clinical Excellence in the United Kingdom (NICE) has recently released guidelines on the treatment of osteoarthritis that also follow a hierarchical approach, combining pharmacological and non-pharmacological treatments (NICE, 2008).

Of interest in this paper is the use of pharmacological preparations for pain relief and whether GPs prescribe in a stepwise manner, or whether they prescribe in line with the level of pain intensity reported by the patient. The latter approach would be more consistent with the British National Formulary (BNF)'s (Joint Formulary Committee, 2011) classification of medications. The BNF describes drugs in terms of their pharmacological classification: non-opioid, opioid and non-steroidal anti-inflammatory (NSAID). Within the opioid and non-opioid groups, the severity of pain that each drug might be used to treat is described as mild, moderate or severe.

The Prognostic Research (PROG-RES) study (Mallen et al., 2006) of primary care consulters with musculoskeletal pain provides the opportunity to investigate, in a natural setting, the approach that GPs use when prescribing medication for musculoskeletal pain.

## 2. Methods

### 2.1 Study sample

Older patients (≥50 years) were eligible for enrolment into the PROG-RES study if they consulted their GP with non-inflammatory musculoskeletal pain during the recruitment period (from September 2006 to March 2007). GPs collected information on the ‘index’ site and intensity of pain during the consultation (Mallen et al., 2006). Within 1 week of this ‘index’ consultation, patients were sent a postal questionnaire, which collected information on socio-demographics, pain, function and other general health constructs. The questionnaire also included a request for written informed consent to access medical records. Data from this questionnaire were not used directly in the analyses presented in this paper.

GPs identified 650 people consulting with musculoskeletal pain, of whom 502 responded to the baseline questionnaire and 428 of these gave permission for their medical record to be accessed. These people made up the sample for use in this paper. Comparing this group with the original 650 people, there was no evidence of response bias with respect to gender. However, those aged 70–79 years were slightly over-represented compared with those who had consulted their GP (Mallen, 2009).

Ethical approval was obtained from the Central Cheshire Local Research Ethics Committee (06/Q1503/60).

### 2.2 Outcome of interest

**Figure 1:**
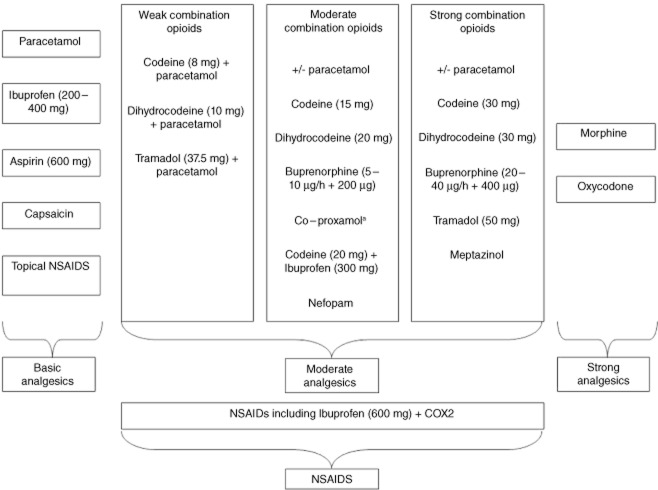
Drug classification hierarchy. Adapted from Bedson et al. (2010). ^a^Combination of paracetamol and dextropropoxyphene.

In those consenting to medical record review, prescriptions for analgesics and NSAIDs were identified on the day of the index consultation. Prescriptions for analgesics were grouped according to an adapted classification of the criteria of Bedson et al. (2010) as mild, moderate or strong analgesics, or an NSAID, which, although having analgesic effects, were considered a separate group to general analgesics because of their anti-inflammatory properties (Fig. 1). This classification takes into account the fact that the full dose of an analgesic in a group will have been used before using the next level of analgesia in the categorization to achieve pain control. Where a person received a prescription in more than one analgesic category on the day of the index consultation, the prescription in the strongest category was used in the analysis.

### 2.3 Predictors of interest

Pain intensity at the index pain site on the day of the index consultation was reported by the patient on a 0–10 numerical rating scale. This was recorded on an electronic template by the GP (Mallen et al., 2006). The scale was grouped into three categories, as has been recommended previously (Serlin et al., 1995; Turner et al., 2004): mild (1–4), moderate (5–6) and severe (7–10). A score of 0 was considered to represent no pain.

Prescriptions for analgesic medications were identified in 12 months prior to the index date and were classified according to the adapted criteria of Bedson et al. (2010). Where a person had received more than one such prescription in the 12-month period, the most recent prescription was used in the analysis. Similarly, NSAID prescriptions in the previous 12 months were also identified.

### 2.4 Statistical analysis

Pain intensity group was entered as the dependent variable into a partial proportional odds model to assess its association with the strength of analgesic prescription on the day of the index consultation (none, basic and moderate/strong). Proportionality of odds was assessed using Wald tests (Williams, 2006). Similarly, the association of pain intensity with the prescription of an NSAID was assessed using binary logistic regression. Adjustment was made for age, gender, most recent analgesic prescription in the previous 12 months and the prescription of an NSAID in the previous 12 months.

## 3. Results

The mean age of the sample was 65 years (standard deviation 10 years) and 60% were female (Table 1). Approximately half had pain at the index site for less than three months and around 65% had pain only at this site. The distribution of pain intensity in the consultation was spread across the three groups (mild, moderate and severe). One person reported a pain intensity score of 0 and was excluded from further analyses.

**Table 1:** Baseline characteristics of sample (*n* = 428)

Characteristic	*n* (%)
Mean age (SD) (years)	65.1 (10.2)
Gender^a^	
Female	255 (59.9)
Male	171 (40.1)
Duration of current pain^a^	
Less than 3 months	212 (50.7)
3–6 months	64 (15.3)
7–12 months	44 (10.5)
1–3 years	45 (10.8)
More than 3 years	53 (12.7)
Pain outside index site^a^	
No	251 (65.4)
Yes	133 (34.6)
Pain intensity group^a^,^b^	
Mild (1–4)	96 (23.9)
Moderate (5–6)	133 (33.1)
Severe (7–10)	173 (43.0)
Analgesic group most recently prescribed in 12 months prior to index consultation	
No prescription	161 (37.6)
Basic analgesics	80 (18.7)
Moderate analgesics	184 (43.0)
Strong analgesics	3 (0.7)
NSAID prescribed in 12 months before index consultation	53 (12.4)
Strongest analgesic group prescribed on day of index consultation	
No prescription	309 (72.2)
Basic analgesics	34 (7.9)
Moderate analgesics	85 (19.9)
Strong analgesics	0 (0.0)
NSAID prescribed on day of index consultation	43 (10.1)

NSAID, non-steroidal anti-inflammatory drug; SD, standard deviation.

a Subject to missing data.

b One person reported pain intensity at index consultation of 0. This person was excluded from analyses.

The majority of patients had received at least one prescription for an analgesic in 12 months before the index consultation (62%), but not on the day of the index consultation (72%). Nobody received a prescription for a strong analgesic on the day of the index consultation.

Overall, 49% of people received the same strength of prescription on the day of the index consultation as they had received previously, while 43% received a prescription of a higher strength. The reporting of higher intensity pain was associated with approximately double the odds of receiving a prescription for an analgesic and for this analgesic to be of a higher strength on the day of the index consultation (Table 2). After adjustment for most recent analgesic prescription in the previous 12 months, this association was attenuated and the strength of previous analgesic prescription was significantly associated with the likelihood of receiving an analgesic prescription on the day of the index consultation and with the strength of this prescription. Adjustment for an NSAID prescription in the previous year, age group and gender made little difference to these associations. The assumption of proportional odds was met in all models.

**Table 2:** Association of previous analgesic prescription and pain intensity on day of index consultation with analgesic prescription at index consultation

	Strength of analgesic prescribed (OR, 95% CI)		
	
	Unadjusted	Adjusted for previous analgesic prescription	Adjusted for previous analgesic, previous NSAID, age and gender
Pain intensity			
Mild	1	1	1
Moderate	1.96 (1.05, 3.66)	1.40 (0.72, 2.69)	1.41 (0.72, 2.75)
Severe	2.09 (1.15, 3.82)	1.44 (0.76, 2.71)	1.49 (0.78, 2.84)
Strength of analgesic prescription in previous 12 months			
None	–	1	1
Mild		3.86 (1.96, 7.58)	3.24 (1.58, 6.61)
Moderate/Strong		2.51 (2.50, 8.14)	4.03 (2.19, 7.42)
NSAID in previous 12 months	–	–	
No			1
Yes			1.17 (0.82, 2.23)
Age group			
50–64 years	–	–	1
65 years and over			1.39 (0.87, 2.25)
Gender			
Female	–	–	1
Male			1.01 (0.63, 1.61)

CI, confidence interval; NSAID, non-steroidal anti-inflammatory drug; OR, odds ratio.

**Table 3:** Association of previous analgesic prescription and pain intensity on day of index consultation with NSAID prescription at index consultation

	NSAID prescribed (OR, 95% CI)		
	
	Unadjusted	Adjusted for previous NSAID prescription	Adjusted for previous analgesic, previous NSAID, age and gender
Pain intensity			
Mild	1	1	1
Moderate	1.50 (0.58, 3.86)	0.95 (0.35, 2.59)	1.13 (0.39, 3.32)
Severe	1.66 (0.68, 4.09)	1.24 (0.49, 3.14)	1.57 (0.57, 4.31)
NSAID in previous 12 months	–	5.60 (2.62, 11.96)	6.34 (2.83, 14.22)
Strength of analgesic prescription in previous 12 months			
None	–	–	1
Mild			1.50 (0.55, 4.13)
Moderate/Strong			0.84 (0.35, 2.03)
Age group			
50–64 years	–	–	1
65 years and over			0.80 (0.39, 1.66)
Gender			
Female	–	–	1
Male			1.37 (0.68, 2.78)

NSAID, non-steroidal anti-inflammatory drug.

There was no significant association between the reported intensity of pain and the prescription of an NSAID on the day of the index consultation (Table 3). However, those people prescribed an NSAID in the previous 12 months had almost six times the odds of such a prescription on the day of the index consultation compared with those who had not received such a prescription previously. Adjustment for the strength of previous analgesic prescription, age group and gender did not alter these associations.

## 4. Discussion

The majority of patients did not receive a prescription for either an analgesic or for an NSAID on the day of their index consultation, although a small proportion received both types of medication and some received prescriptions for more than one strength of analgesic. There was an association between the intensity of pain reported by the patient and the strength of analgesic prescribed by the GP, but this was attenuated by adjustment for previous prescriptions. The majority of patients received a prescription on the day of the index consultation that was of the same strength or one group stronger than they had received previously. There was no association between the reported intensity of pain and the prescription of an NSAID. As with analgesics, the strongest predictor of an NSAID prescription was having received such a prescription in the last 12 months.

These data have the unique advantage that pain intensity was recorded by the GP during routine, primary care consultations. As a consequence, the GP's record of pain intensity is likely to be both accurate and contemporaneous.

All prescriptions in UK primary care are recorded routinely; hence, all prescriptions have been captured. However, many pain-relief medications can be bought over the counter (OTC) in the United Kingdom, and for those people who have to pay for prescribed medication (£7.40 in 2011), purchasing OTC will often be a cheaper option for the patient. The usage of drugs available OTC (mainly basic analgesics) will therefore be underestimated, especially in those aged under 60 years who are required to pay the prescription charge in England. Furthermore, even when considering prescribed medication, there is evidence that up to 5% of patients do not collect prescriptions from the pharmacy and that in those that do, up to 50% do not adhere to the suggested treatment regimen (Garfield et al., 2009).

What is not clear from this study is whether patients had previously received treatment either pharmacological or non-pharmacological for their pain (at the index site or another) that could have reduced the level of pain intensity reported. This was to an extent overcome by adjustment for previous prescriptions. However, this does not directly assess the reduction in pain intensity achieved by previous treatment or whether patients had used OTC analgesia or potentially accessed non-pharmacological treatment.

There have been several suggestions in the literature as to the appropriate way in which to group a 0–10 numerical rating scale for pain intensity into mild, moderate and severe groups. The grouping used in this study was chosen as it has been widely advocated and shown to be plausible in a range of patient groups (Serlin et al., (1995) – cancer pain, Jensen et al. (#b^1001^) – low back pain).

A previous study in Austria showed no association between the reported pain intensity and the type of analgesic prescribed (Tönies and Maier, 2001). The authors ascribed this finding to the fact that their sample was not restricted to those patients with pain, meaning some analgesics could have been prescribed for other reasons (e.g. feverish illness). The difference in findings between the current study and that of Tönies and Maier (2001) could be explained by the restriction of the PROG-RES sample to patients with (non-inflammatory) musculoskeletal pain. Hence, the current study represents a more appropriate group in which to study this association. It is possible that patients in the PROG-RES study received analgesics, particularly the stronger preparations, for other conditions such as cancer, but previous research has shown that the majority of pain complaints are musculoskeletal in origin (McBeth and Jones, 2007).

This study provides evidence that patients reporting more intense pain are more likely to receive a prescription for an analgesic from their GP and that the strength of that analgesic is likely to be higher. However, the main driver for the prescription decision appears to be previous prescription. This indicates that while GPs prescribing practice may appear to follow the rubric of the BNF description of medications, GPs actually adopt an individualized approach to their treatment of patients' musculoskeletal pain that incrementally increases treatment, more in line with the WHO guidelines on cancer pain (WHO, 1986), or the NICE guidelines for the treatment of osteoarthritis (NICE, 2008), which were issued after the data used in this study were collected. To put this in context, it seems that GPs take into account a range of factors, including pain intensity, patient pressure, health economics and adverse outcomes before prescribing (Lanza et al., 2009). This is especially true when considering NSAIDs, where side effect profile is often more toxic (Schaffer et al., 2006, Thompson et al., 2005).

The lack of a prescription in the majority of cases, particularly in those with mild pain, suggests that GPs may be recommending non-pharmacological treatments or OTC medication for musculoskeletal pain. Hence, GPs appear to be taking a pragmatic and individualized approach to the treatment of musculoskeletal pain in older adults. This might incorporate a ‘step wise’ use of analgesics as suggested by guidelines, but an individual's health needs and personal risks influence the GP's choices. For example, an elderly patient at risk of falling might not be given the strongest opioid analgesic they might require to alleviate their pain as suggested by the next step in the NICE OA guidelines (NICE, 2008), since the potential side effects might increase the risk of such a fall and result in a fractured hip (Saunders et al., 2010). This evidence is strengthened by the knowledge that patients in the United Kingdom are generally able to see the same GP on each visit to the practice, and so receive continuity of care. Taken as a whole, the evidence suggests that GPs are acting in line with guidance such as that from the European League Against Rheumatism recommendations for knee osteoarthritis (Pendleton et al., 2000), where an individualized approach is advocated.

This study has provided information on the patterns of the prescribing of analgesics and NSAID to patients aged 50 years and over consulting in UK primary care, relative to pain intensity and previous prescriptions. What it cannot tell us though is the reason for the issue of these prescriptions and the thought processes behind these prescribing decisions. Further work, in a larger sample, might consider new episodes of care so as to separate newly prescribed preparations from repeat prescriptions and also account for non-pharmacological treatments.

This study has suggested that GPs do not prescribe analgesia for musculoskeletal pain as a matter of course. When prescriptions are issued, the severity of a patient's pain appears to be associated with the strength of prescribed analgesic, but this is superseded by previous prescriptions.

## Author contributions

All authors were involved in drafting the paper or revising it critically for important intellectual content, and all authors approved the final version to be submitted for publication. Dr. Muller had full access to all of the data in the study and takes responsibility for the integrity of the data and the accuracy of the data analysis.

Study conception and design: S. Muller, J. Bedson and C.D. Mallen.

Acquisition of data: C.D. Mallen.

Analysis and interpretation of data: S. Muller, J. Bedson and C.D. Mallen.
